# A Case of Ovarian Pregnancy Diagnosed by MRI

**DOI:** 10.1155/2015/143031

**Published:** 2015-09-27

**Authors:** Shingo Io, Masaaki Hasegawa, Takashi Koyama

**Affiliations:** ^1^Department of Gynecology and Obstetrics, Kyoto University, 54 Kawahara-cho, Shogoin, Sakyo-ku, Kyoto 606-8507, Japan; ^2^Department of Obstetrics and Gynecology, Kurashiki Central Hospital, Japan; ^3^Department of Diagnostic Radiology, Kurashiki Central Hospital, Japan

## Abstract

Ovarian pregnancy is a rare form of ectopic pregnancy, causing a great diagnostic challenge. We report a case of ovarian pregnancy in a 42-year-old woman, in whom MRI successfully demonstrated the implantation in the ovary. Transvaginal ultrasonography showed an echogenic mass in the right ovary but failed to demonstrate tubal pregnancy. T2-weighted MR images disclosed a gestational sac structure in the right ovary, which exhibited heterogeneous high intensity intermingled with punctate foci of distinct low intensity. MRI may be a useful tool for diagnosing ovarian pregnancy, by demonstrating a gestational sac in the ovary.

## 1. Introduction

Ovarian pregnancy is a rare event with an estimated incidence of 1% to 6% of all ectopic pregnancies [1–4]. The distinction of ovarian pregnancy from the much more common ectopic pregnancy occurring in the fallopian tube usually depends on findings from transvaginal ultrasonography (TV-US). Reported TV-US features in ovarian pregnancy include a cyst on the ovary with a wide echogenic outside ring, fluid collection surrounding the ovary, and an absence of hematosalpinx [1, 2, 5, 6]. However, this condition is still a diagnostic challenge, and laparoscopy is usually required for the diagnosis [2]. Magnetic resonance imaging (MRI) has served as a problem-solving modality in ectopic pregnancies by providing excellent tissue contrast for an implantation site, even when it is unclear on TV-US [7–12]. As far as we know, there are no reports concerning the MRI findings of ovarian pregnancy. Hereby, we report MRI findings in a case of ovarian pregnancy.

## 2. Case Presentation

A 42-year-old woman, gravida 1, para 1, presented to her local clinic complaining of amenorrhea for six weeks with a positive pregnancy test. She did not complain of any abdominal pains and vaginal bleeding. She had a history of delivery by cesarean section 5 years ago. She did not have any past history of pelvic inflammatory disease or insertion of an intrauterine device.

**Figure 1:**
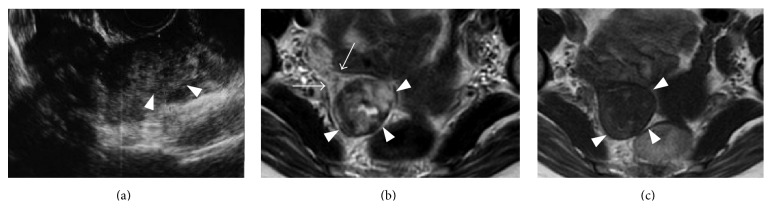
(a) Transvaginal ultrasonography reveals an echogenic mass (arrowhead) incarcerated to the right ovary and a normal endometrium. (b) Axial T2-weighted MR image shows a GS structure of heterogeneous high intensity (arrowhead), containing punctate foci of distinct low intensity. The GS is incarcerated to the right ovary, forming a “beak sign” (arrows). (c) Axial T1-weighted MR image showed GS structure (arrowhead) containing punctate foci of high intensity.

TV-US performed at her local clinic showed an empty uterine cavity and normal adnexa size at sixth week. At eighth week, TV-US revealed hematoma on the right adnexa, and she was referred to our hospital with suspected ectopic pregnancy based on a serum beta human chorionic gonadotropin level of 16,265 mIU/mL. A serum alpha-fetoprotein level was within normal limit. TV-US in our hospital showed an echogenic mass in the right enlarged ovary (Figure 1(a)) and a normal endometrium. Color Doppler US revealed blood flow in the mass. However, we could not exclude tubal pregnancy, since the right fallopian tube was poorly seen.

Pelvic MRI was performed for the purpose of precise localization of the implantation site. T2-weighted MR images disclosed a gestational sac (GS) structure, which exhibited a heterogeneous high intensity intermingled with punctate foci of distinct low intensity on T2-weighted images, incarcerated to the posterior surface of the right ovary (Figure 1(b)). The mass formed a “beak sign” in the ovary [13], in the absence of a dilated fallopian tube. T1-weighted images revealed foci of high intensity in the mass (Figure 1(c)), corresponding to the low intensity on T2-weighted images, suggesting hemorrhage.

**Figure 2:**
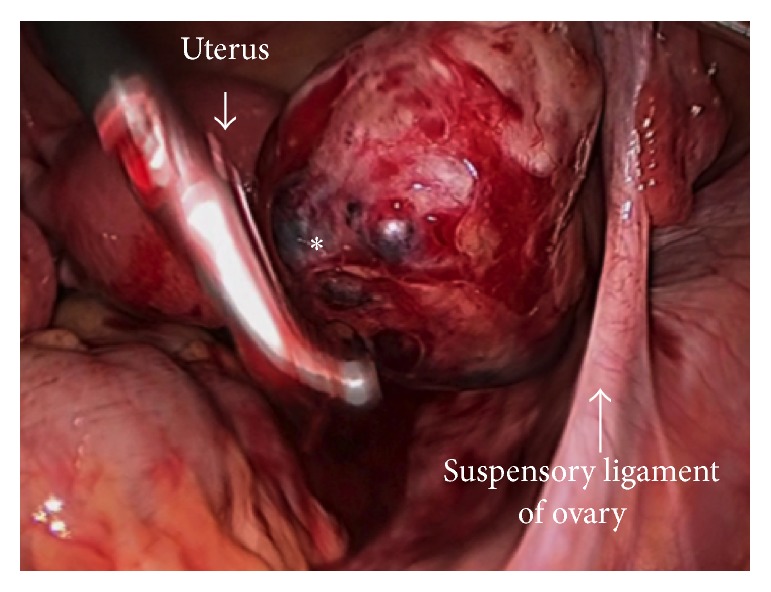
Laparoscopic image demonstrates an ectopic GS (*∗*) incarcerated to the posterior surface of the right ovary.

**Figure 3:**
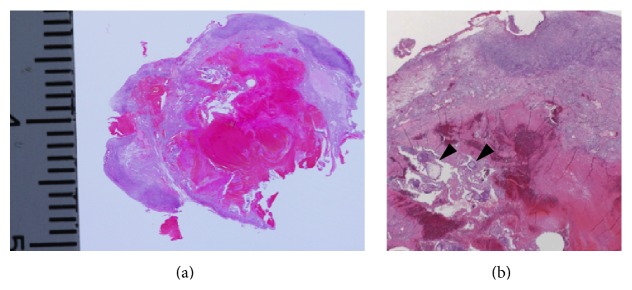
(a) Postoperative pathology, corresponding to the MR image, illustrates chorionic villous structures surrounded by ovarian stroma. (b) Photomicrograph shows chorionic villous structures (arrowhead) (H&E stain, 40x magnification).

Subsequently performed laparoscopy revealed an unruptured right ovarian pregnancy, with a GS structure attached to the posterior surface of the ovary (Figure 2). Unilateral salpingooophorectomy was performed on the right side, because she did not desire to bear any further children and prefer salpingooophorectomy rather than ovarian wedge resection. Postoperative pathological analysis confirmed right ovarian gestation, demonstrating both chorionic villi and trophoblasts, and neighboring corpus luteum (Figure 3). The patient had an uneventful postoperative period and was discharged without complications.

## 3. Discussion

TV-US is a highly accurate modality for the diagnosis of ectopic pregnancies [8, 14, 15]. The most important TV-US finding indicating tubal pregnancy is an adnexal mass that is distinguishable from the ovary [16]. The tubal ring sign, which is an echogenic ring surrounding an extrauterine GS, is also known as the second most common sign of tubal pregnancy [16]. However, TV-US may occasionally fail to detect extrauterine GS in the presence of tubal hematoma or hemoperitoneum, and extrauterine GS can mimic corpus luteum cysts or theca lutein cysts [8].

On the other hand, the diagnosis of ovarian pregnancy by TV-US is difficult. Choi et al. suggested that the rate of accurate preoperative diagnosis of ovarian pregnancy by TV-US examination was only 18% [2]. In the current case, TV-US revealed an echogenic mass in the enlarged ovary. It was suggested that the mass, which was inseparable from the ovary, was an atypical feature for tubal pregnancy. We were unconvinced of ovarian pregnancy, because it is rare and the fallopian tube was poorly seen.

MRI is useful tool for accurately localizing the implantation site, especially when TV-US findings are insufficient or equivocal [7–12]. A MRI finding indicating an ectopic pregnancy is the presence of extrauterine GS structures that typically appear as mass high intensity containing foci of distinct low intensity on T2-weighted images which represent hemorrhage. In tubal pregnancy, the recognition of wall enhancement of dilated tubal structure is another important finding to indicate tubal pregnancy. In our case, MRI successfully demonstrated a GS structure incarcerated to the ovary.

An important differential diagnosis of this condition is corpus luteum, which is frequently associated with pregnancy. Different from a GS, corpus luteum cysts usually have a thin wall that shows slightly increased intensity on T1-weighted images and that does not contain acute hematomas of distinct low intensity on T2-weighted images [8].

In conclusion, when findings on TV-US are inconclusive for suspecting ovarian pregnancy, MRI may be a useful tool for diagnosing ovarian pregnancy, by demonstrating a gestational sac in the ovary.
